# Clinical, Molecular Characteristics, and Genotype–Phenotype Relationships of Metaphyseal Chondrodysplasia Type Schmid

**DOI:** 10.1007/s00223-025-01457-8

**Published:** 2025-12-27

**Authors:** Lingyang Meng, Jing Hu, Lei Sun, Qian Zhang, Ou Wang, Yan Jiang, Xiaoping Xing, Weibo Xia, Mei Li

**Affiliations:** Department of Endocrinology, Key Laboratory of Endocrinology of National Health Commission, Peking Union Medical College Hospital, Chinese Academy of Medical Sciences and Peking Union Medical College, Shuaifuyuan No. 1, Dongcheng District, Beijing, 100730 China

**Keywords:** Metaphyseal chondrodysplasia type Schmid, *COL10A1*, Type X collagen, Genotype–phenotype relationships

## Abstract

Metaphyseal chondrodysplasia type Schmid (MCDS), a rare skeletal disorder caused by *COL10A1* mutations, exhibits significant phenotypic heterogeneity, yet genotype–phenotype correlations remain poorly defined. We aim to determine the clinical and radiographic manifestation, mutational features, and genotype–phenotype relationships by characterization of 4 patients with MCDS and literature review. The four patients presented with short stature or waddling gait, flattened vertebrae, and irregular femoral epiphyses. We identified two novel *COL10A1* variants (c.1925 T > A, c.1903C > G) and reported the first case harboring both a de novo nonsense (c.2001 T > G, p.Tyr667Ter) in the non-collagenous 1 (NC1) domain and a missense (c.1438A > T, p.Ile480Leu) in the helix domain. Genotype–phenotype analysis of 124 cases previously reported and 4 new cases revealed that NC1 domain mutations were associated with an earlier onset of MCDS than non-NC1 mutations (median 12 vs. 72 months, *P* = 0.0014). Patients carrying a missense mutation in *COL10A1* showed significantly lower height Z-scores (− 3.62 ± 1.95 vs. − 1.99 ± 1.28, *P* = 0.013) at first and more metaphyseal irregularities in the distal radius/ulna than those with truncating mutations (*P* = 0.019). Structural modeling indicated that NC1 mutations may disrupt collagen X structure via electrostatic alterations or steric clashes. These findings expand the mutational spectrum of MCDS and establish that *COL10A1* genotype correlates with severity of MCDS, which will help to identify patients with severe phenotypes through molecular testing and to develop effective treatment strategies for MCDS.

**Supplementary Information:**

The online version contains supplementary material available at 10.1007/s00223-025-01457-8.

## Introduction

Metaphyseal chondrodysplasia type Schmid (MCDS, OMIM 156500), an extremely rare autosomal dominant hereditary disease with a prevalence of approximately 3–6 per million [1], is characterized by short stature, waddling gait, limb deformity, and metaphyseal irregularities, including widening, fraying, and cupping in X-ray films, typically presenting in childhood [2, 3]. MCDS is caused by mutations in the *COL10A1* gene, which encodes the α1 chain of type X collagen, a homotrimer of three α1 chains [4]. Collagen X, exclusively expressed by the hypertrophic chondrocytes in the growth plate cartilage [4], contains signal peptide, non-collagenous 1 (NC1) domain at the C-terminal, triple helix domain, and NC2 domain at the N-terminal [5], and assembles into a supramolecular hexagonal lattice within the cartilage matrix, functioning in fetal chondrogenesis and endochondral ossification [6, 7].

The diagnosis of MCDS is based on clinical, radiologic features, and molecular testing. However, MCDS is prone to being misdiagnosed as rickets due to the possibility of causing lower limb bending deformities [8–10]. Additionally, effective treatment for MCDS is lacking, and treatment is mainly supportive, such as osteotomy [1]. Previous studies have mostly focused on case reports or a few family studies, and relatively large cohorts are needed to elucidate the genotype–phenotype relationships and to develop effective diagnostic and treatment strategies for MCDS.

In this study, we investigate the clinical, radiographic manifestations, and molecular features of a relatively large cohort of MCDS. We also review the molecular and phenotypic findings of previously reported MCDS patients to improve the understanding of diagnosis and treatment strategies for MCDS.

## Materials and Methods

### Patients

Patients from different nonconsanguineous families were included in the Department of Endocrinology at Peking Union Medical College Hospital (PUMCH) between 2019 and 2023. They were clinically suspected of metaphyseal dysplasia because of short stature or metaphyseal abnormalities in the spine or lower limbs shown on the radiography.

This study was approved by the ethics committee of PUMCH (JS-2081), and informed consents were signed by parents or legal guardians of all patients.

### Clinical and Radiographic Evaluation

Height and weight Z-scores of the patients were derived according to growth reference data of Chinese children and adolescents aged 0–18 years [11].

Fasting blood samples were collected, and serum calcium, phosphate, alkaline phosphatase (ALP), growth hormone (GH), insulin-like growth factor 1 (IGF-1), alanine aminotransferase (ALT), and creatinine (Cr) were measured using an automatic biochemical analyzer (ADVIA 1800, Siemens, Germany). Serum concentrations of parathyroid hormone (PTH), 25-hydroxyvitamin D (25-OHD), β-isomerized C-terminal telopeptide of type I collagen (β-CTX, biomarker of bone resorption), and procollagen type 1 aminoterminal peptide (P1NP, biomarker of bone formation) were detected by an automated electrochemiluminescence system (Roche Diagnostics, Switzerland).

Radiographs of the spine, pelvis, hand, and lower limbs were obtained at baseline and follow-up. Dual-energy X-ray absorptiometry (Lunar Prodigy, GE Healthcare, Madison, WI, USA) was used to measure bone mineral density (BMD) at lumbar spine 1–4 (LS), femoral neck (FN), trochanter (TR), and total hip (TH) at baseline and follow-up. The Z-scores of BMD were obtained according to the age- and sex-matched normal ranges of Chinese or Asian children [12, 13].

### Genetic Mutation Identification

Genomic DNA was extracted from peripheral blood leukocytes using a QIAamp DNA Blood Midi/Mini kit (QIAGEN GmbH, Hilden, Germany). A DNA library was prepared using NanoWES Human Exome V2.0 (Berry Genomics, China) according to the manufacturer’s protocol. Whole-exome sequencing (WES) was completed by Novaseq 6000 platform (Illumina, San Diego, United States). The sequencing reads were aligned to the human reference genome (hg19/GRCh37), and PCR duplicates were removed by Picard v1.57. Variant calling was performed with Verita Trekker Variants Detection System and the software GATK. All variants were filtered through population databases, including gnomAD, the 1000 Genomes Project (1000G), and Exome Aggregation Consortium (ExAC). Only the variants with population frequencies less than 1/1000 in all databases were counted. The variants were classified according to the 2015 American College of Medical Genetics and Genomics and Association for Molecular Pathology (ACMG/AMP) Standards and Guidelines [14]. The *COL10A1* variants identified by WES were confirmed by PCR and Sanger sequencing, of which the primer sequences are shown in supplemental materials and methods [15].

### In Silico Analysis of *COL10A1* Mutations

Sequence alignments of *COL10A1* mutation sites in our patients were performed on the Clustal Omega website. Chimera Ⅹ 1.9 was used to build the model of the NC1 domain of type X collagen (Protein Data Bank entry 1GR3) and to predict the electrostatic potential changes and spatial clashes of mutant amino acids.

### Literature Review

We conducted a literature review by searching for studies or case reports of MCDS published from 1993 to May 2025 on PubMed, Web of Science, and Google Scholar databases, using the following search string: (“*COL10A1*” OR “type X collagen” OR “collagen X”) AND (metaphyseal chondrodysplasia type Schmid).

### Statistical Analyses

Categorical variables, such as genotype classification, signs, and clinical and radiographic features, were presented as frequencies and percentages. Normality of continuous variables was assessed by Shapiro–Wilk test. Continuous variables with normal distribution were presented as mean ± SD, while those that were non-normally distributed were presented as median (interquartile range). Mann–Whitney U test or unpaired t-test was used to compare ages and height Z-scores between different genotype groups. Comparison of the percentage of clinical and radiographic features between patients with different genotypes was performed using Fisher’s exact test.

A *P* value < 0.05 was considered statistically significant. The above analyses were performed with GraphPad Prism 9.4.1.

## Results

### Clinical Manifestations and Treatment of the Patients

4 children from unrelated families were initially diagnosed with spondyloepiphyseal dysplasia, skeletal deformity, or short stature in PUMCH (Table 1).

**Table 1:** Clinical characteristics of patients with MCDS in PUMCH

	Patient No.			
	1	2	3	4
Gender	M	M	F	M
Age of initial sign (y)	6–7	1.1	0	2
Initial sign	Waddling gait	Bowed legs	Waddling gait	Short stature
Age of first Dx (y)	10.1	1.6	6.8	5.3
First Dx	Spondyloepiphyseal dysplasia	Skeletal deformity	Skeletal deformity	Short stature
Final Dx	MCDS	MCDS	MCDS	MCDS
Family history	No	No	Mother, maternal grandmother	No
Follow-up (y)	2.9	6.1	1.1	2.4
Bone pain	No	No	Yes	No
Fracture	No	No	No	No
Shorten limb	No	No	No	No
Waddling gait	Yes	No	Yes	No
Bowed legs	No	Yes	No	No
Short stature	No	Yes	No	Yes
Height (cm)/Z-score at first Ex	139/− 0.2	–	113/− 1.9	97/− 3.9
Height (cm)/Z-score at last Ex	159.5/0.0	106/− 4.4	119.5/− 1.7	110.6/− 3.5
Weight (kg)/Z-score at first Ex	32/− 0.2	–	29/1.8	10.3/− 3.6
Weight (kg)/Z-score at last Ex	56/0.7	–	37/2.7	18.5/− 2.2
Radiologic features of the spine	Irregular and flat vertebrae, anterior displacement of L3 and L5 vertebrae, decreased vertebral BMD, blurred trabeculae	Flat vertebrae, lumbar lordosis	Decreased vertebral BMD, lumbar lordosis, slight flattening of the T7 vertebrae	Long pedicles
Radiologic features of the lower limb	Femoral neck shortening, widening of proximal femoral epiphyses	Widening of femoral and tibiofibular epiphyses with irregular pattern	Slight widening of femoral and tibial epiphyses, coxa vara, slightly bowed femurs, subluxation of the hip joint	No abnormality

	Patient No				Reference range for children
	1	2	3	4	
Radiologic features of the hand	No abnormality	Brush change of the distal metaphyses of the radius and ulna, slight thickening of metacarpals and phalanges	No abnormality	No abnormality	NA
LS BMD (g/cm^2^)/Z-score	0.761/1.7	–	0.784/2.7	0.591/1.2^a^	NA
FN BMD (g/cm^2^)/Z-score	0.759/0.3	–	0.727/1.1	0.639/− 0.2^a^	NA
TR BMD (g/cm^2^)	0.631	–	0.559	0.502^a^	NA
TH BMD (g/cm^2^)	0.759	–	0.747	0.685^a^	NA
Ca (mmol/L)	2.61	2.54	2.50	2.59^a^	2.13–2.70
P (mmol/L)	2.04	1.57	1.64	1.52^a^	0.95–2.65
β-CTX (ng/mL)	1.49	1.31	1.86	2.13^a^	0.40–3.30
P1NP (ng/mL)	439.0	953.4	664.0	589.0^a^	30.0–3000
PTH (pg/mL)	25.2	18.8	30.9	23.1^a^	15.0–65.0
25OHD (ng/mL)	23.0	27.7	18.2	50.9^a^	Deficiency: < 20; Insufficiency: 20–30; Sufficiency: > 30
ALP (U/L)	580	415	390	202	42–390
ALT (U/L)	29	18	17	13^b^	9–50
Cr (μmol/L)	44	23	31	44^b^	45–84
GH (ng/mL)	–	0.3	< 0.05	0.5^b^	< 2.0
IGF-1 (ng/mL)	–	88	234	110^b^	57–277

^a^Data at the 13-month follow-up

^b^Data at the 7-month follow-up

25OHD, 25-hydroxyvitamin D; ALT, alanine aminotransferase; BMD, bone mineral density; β-CTX, cross-linked C-telopeptide of type I collagen; Ca, serum calcium; Cr, creatinine; Dx, diagnosis; Ex, examination; FN, femoral neck; GH, growth hormone; IGF-1, insulin-like growth factor 1; LS, lumbar spine; MCDS, metaphyseal chondrodysplasia type Schmid; NA, not applicable; P, serum phosphate; P1NP, procollagen type 1 amino-terminal peptide; PTH, parathyroid hormone; TH, total hip; TR, trochanter

**Fig. 1:**
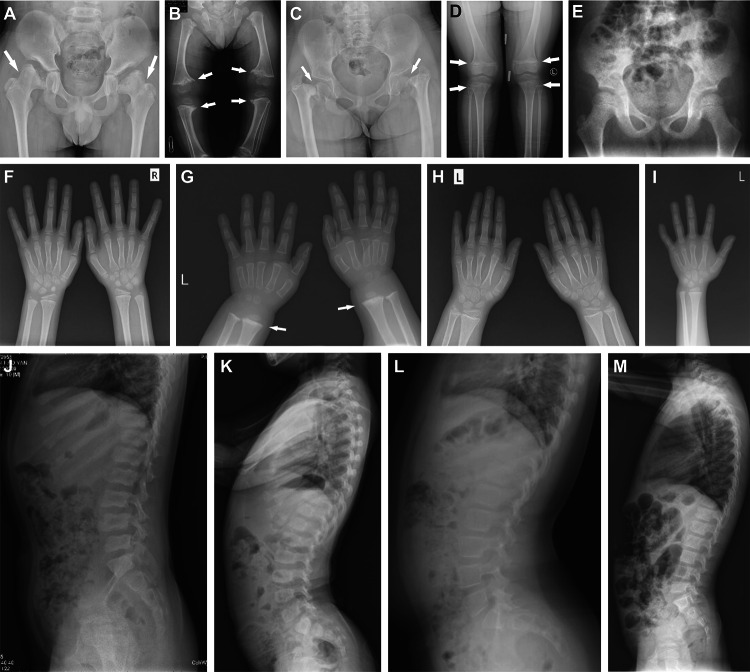
Radiographic features of 4 patients with MCDS in PUMCH. **A**, **F**, **J** Radiographs of pelvis, hand, and thoracolumbar spine of patient 1. The arrows indicate widening of the femoral epiphysis and a short femoral neck. The vertebrae were irregular and flat. The distal ulnar and radial epiphyses were normal. **B**, **G**, **K** Radiographs of the lower extremities, hand, and spine of patient 2. Arrows indicate metaphyseal widening of distal femurs and proximal tibiae, and brush changes of the distal ulnar and radial metaphyses. The spine showed lumbar lordosis and flat vertebrae. **C**, **D**, **H**, **L** Radiographs of pelvis, knee, hand, and thoracolumbar spine of patient 3. Arrows indicate coxa vara and a slightly enlarged epiphysis of the knee. Lumbar lordosis was obvious. The distal ulnar and radial epiphyses were normal. **E**, **I**, **M** Radiographs of pelvis, hand, and spine of patient 4. The femoral, distal ulnar, and radial epiphyses were normal. The vertebral pedicles were overlong

**Fig. 2:**
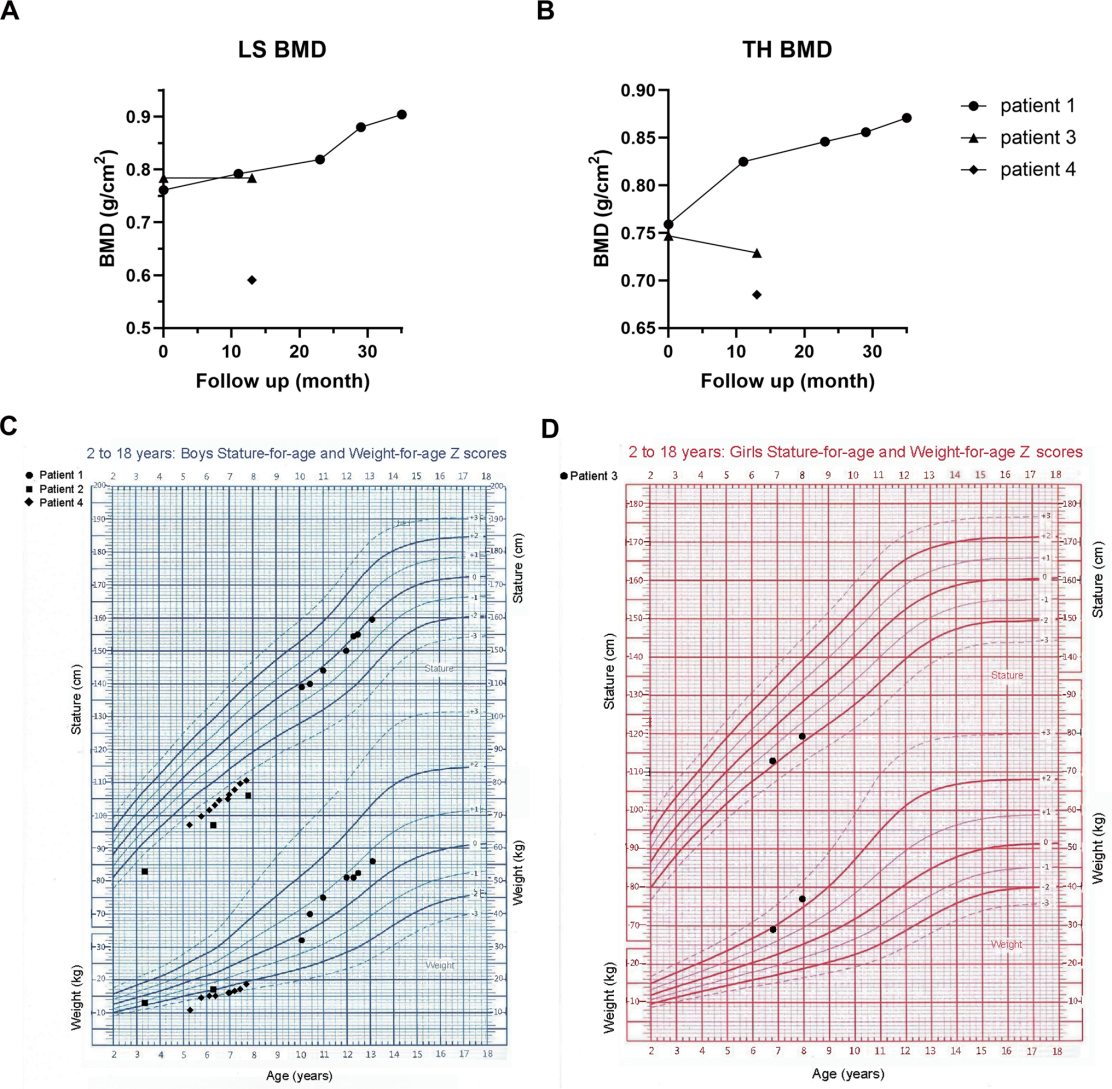
BMD and growth curve of patients with MCDS. **A**, **B** LS and TH BMD changes of patients 1, 3, and 4 during follow-up, respectively. Patient 2 did not undergo a BMD test. LS, lumbar spine. TH, total hip. **C**, **D** Stature Z-score and weight-Z score changes of 4 patients. Patients 2 and 4 showed growth retardation. Only patient 4 received rhGH therapy at his 6.9–7.7 years, but did not show catch-up growth

Patient 1, a 10.1-year-old boy, came to the clinic with a waddling gait for three years. He was the first child in his family and diagnosed with macrosomia with a birth weight of 4.9 kg, and the body length at birth was unknown. He had no bone pain, fracture, shortened limbs, bowed legs, or short stature. The initial diagnosis was spondyloepiphyseal dysplasia due to irregular and flat vertebrae as well as bilaterally flat femoral epiphyses on X-ray films (Fig. 1A and J). No metaphyseal anomaly was found at the metacarpals and phalanges (Fig. 1F). X-rays show decreased bone mineral density in several bones. Family history of skeletal deformity or short stature was denied. Serum Ca, P, β-CTX, P1NP, and PTH levels were all within age-matched normal ranges, but the insufficient 25OHD levels and abnormally elevated ALP levels were found. He was given experimental treatment with 600 mg calcium daily and 0.25 μg calcitriol every other day for 35 months, and alendronate of 70 mg once a week in months 23–29. The LS BMD increased rapidly during alendronate treatment, however, there was no significant improvement in bone deformity or growth speed (Fig. 2A).

Patient 2, a boy aged 1.6 years, manifested bowed legs at 1 year old, and was initially diagnosed as having a skeletal deformity of an unknown reason. At 3.4 years old, he demonstrated a short stature with a height of 83 cm (Z-score − 3.5). He did not suffer from bone pain or fracture, and he had no family history of skeletal deformity or short stature. X-rays showed spinal metaphyseal abnormality, flat vertebrae, lumbar lordosis, and bilaterally wide femoral and tibiofibular epiphyses with an irregular pattern (Fig. 1B and K). The distal ulnar and radial metaphyses also showed brush changes (Fig. 1G). Serum Ca, P, β-CTX, P1NP, PTH, GH, and IGF-1 levels were all within age-matched normal ranges, however, he had insufficient vitamin D. He did not receive medication but underwent bilateral corrective osteotomy for bowed legs at 6.5 years. His growth speed was relatively low, and the heights at the ages of 6.3 and 7.8 years were 97 cm and 106 cm (Z-score − 4.4 for both), respectively (Fig. 2C).

Patient 3, a girl aged 6.8 years, exhibited a waddling gait, genu valgum, and bilateral hip pain, without fracture, shortened limbs, or short stature. Her height was 113 cm (Z-score − 1.9) (Fig. 2D). Her mother and maternal grandmother had similar manifestations. The initial diagnosis was skeletal deformity based on bilateral subluxation of the hip joint, subcapital femoral neck fracture, coxa vara, slightly bowed femurs, lumbar lordosis, and slight flattening of vertebrae shown on the X-rays (Fig. 1C, D, and L). No metaphyseal anomaly was found at the metacarpals and phalanges (Fig. 1H). Serum Ca, P, β-CTX, P1NP, PTH, GH, and IGF-1 levels were all within age-matched normal ranges. She had a vitamin D deficiency and received 300 mg of calcium daily and 0.25 μg of calcitriol every other day for 13 months. After treatment, the height increased to 119.5 cm (Z-score − 1.7), and the LS BMD increased from 0.784 (Z-score 2.7) to 0.816 (Z-score 2.5) g/cm^2^.

Patient 4, a boy aged 5.3 years, mainly manifested short stature, with a height of 97 cm (Z-score − 3.9). He had no bone pain, fracture, or shortened limbs. Additionally, he had a cleft palate, epicanthus, and mild elbow valgus. Family history of short stature was denied. No abnormality was observed on the X-rays of the spine, hip joint, and wrist, except for slight lengthening of the pedicles (Fig. 1E). LS BMD, FN BMD at the 7 months of follow-up, and serum Ca, P, β-CTX, P1NP, PTH, GH, and IGF-1 levels at the 13 months of follow-up were all within age-matched normal ranges. Ultrasonic cardiogram identified ductus arteriosus and patent foramen ovale. He was followed up for 29 months. Experimental treatment of calcium 200 mg plus vitamin D 600 IU was administered daily. To improve the height, recombinant human growth hormone (rhGH) was subcutaneously injected every night at month 20 (height 106.1 cm, Z-score − 3.5 at this time). The dose increased to 0.665 mg sc at month 26. From month 29, the rhGH was administered at 0.831 mg sc. During 9 months of rhGH therapy, the height increased by 4.5 cm (height 110.6 cm, Z-score − 3.5 after therapy), and the growth velocity remained low.

### Genotypes of the Patients

By WES and Sanger sequencing, 5 germline heterozygous *COL10A1* mutations were detected in these 4 patients (Table 2), of which 2 mutations are not reported in the gnomAD database or literature. Hence, the final diagnosis of these patients was corrected as MCDS. Most of the variants affected the NC1 domain of collagen Ⅹ, and the c.1438A > T variant in patient 2 influenced the helix domain of collagen Ⅹ.

**Table 2:** Molecular characteristics of patients in PUMCH with *COL10A1* mutations

Patient No	Nucleotide variant	Protein change	Collagen Ⅹ domain	Novel/Known	Zygosity	Inherited/De novo	ACMG
1	c.2032G > C	p.Ala678Pro	NC1	Known	Heterozygous	Inherited	VUS
2	c.2001T > G	p.Tyr667Ter	NC1	Known	Heterozygous	De novo	P
	c.1438A > T	p.Ile480Leu	Helix	Known	Heterozygous	Inherited	VUS
3	c.1925T > A	p.Ile642Asn	NC1	Novel	Heterozygous	Inherited	VUS
4	c.1903C > G	p.Gln635Glu	NC1	Novel	Heterozygous	NA	VUS

ACMG, American College of Medical Genetics and Genomics; NA, not applicable; NC, non-collagenous; P, pathogenic; Ter, terminator; VUS, variant of uncertain significance

**Fig. 3:**
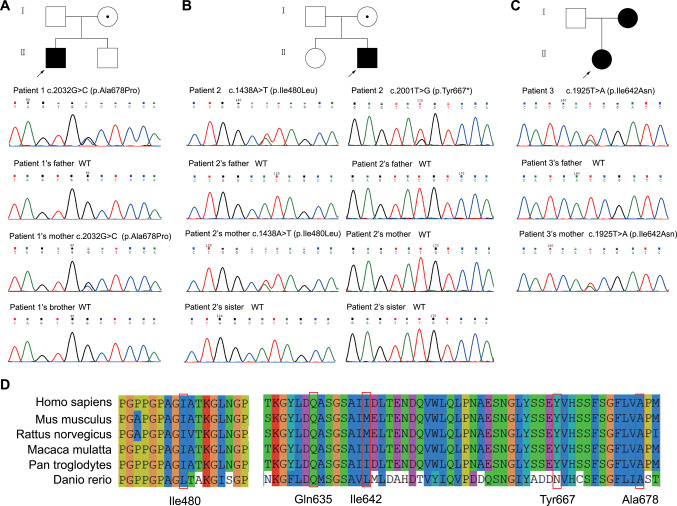
Pedigrees and Sanger sequencing of *COL10A1* mutations in three patients, and sequence conservation at *COL10A1* mutation sites. **A** Patient 1’s pedigree and Sanger sequencing of *COL10A1* mutations. **B** Patient 2’s pedigree and Sanger sequencing of *COL10A1* mutations. **C** Patient 3’s pedigree and Sanger sequencing of *COL10A1* mutations. The arrows indicate probands. The filled symbols represent the affected individual. The hollow symbols with a dot represent asymptomatic mutation-carriers. WT, wild type. **D** Sequence alignment of 5 *COL10A1* mutations from different species

The pedigrees of families 1, 2, and 3 were depicted. Patient 1 carried the c.2032G > C variant (Fig. 3A). His mother was a heterozygous asymptomatic carrier, with a height of 168 cm (Z-score 1.5) and no clinical signs. However, she was unwilling to undergo further imaging examination. His father and brother are wild type. Patient 2 harbored 2 *COL10A1* mutations of the nonsense c.2001T > G (p.Tyr667*) in the NC1 domain and the missense c.1438A > T (p.Ile480Leu) in the helix domain (Fig. 3B). Patient 2’s mother also carried the heterozygous c.1438A > T variant but did not exhibit similar clinical manifestations (height 152 cm, Z-score − 1.7), and no obvious abnormalities were reported on the radiography. His father and sister are wild type. Interestingly, c.2001T > G is a de novo mutation since its absence in both parents, which makes it pathogenic (PVS1 + PS2 + PM2) according to ACMG/AMP Standards and Guidelines. Patient 3 harbored the novel c.1925T > A variant, which was also detected in her mother and maternal grandmother (Figs. 3C and S1 [15]). However, their clinical manifestations were not available. Patient 4 carried the novel c.1903C > G (p.Gln635Glu) mutation, but molecular testing was not performed on his family members, who had normal height (father, 179 cm, Z-score 1.1; mother, 155 cm, Z-score − 1.1; 6-year-old brother, 110 cm, Z-score − 1.6) and no clinical signs. However, they were unwilling to undergo further imaging examination.

Except for site Ile642 (c.1925 T), the amino acids of the remaining 4 mutation loci showed high interspecies conservation (Fig. 3D).

### Structural Analysis of Mutant Collagen Ⅹ

Figure S2A and B illustrated the positions of the altered amino acids by the 4 mutations in the NC1 monomer, as well as their electrostatic potential characteristics [15]. Both Glu635 and Asn642 mutations strengthened the negative potential, with the former being more pronounced. Ter667 resulted in the absence of a peptide segment from the NC1 domain. Although the Pro678 variant did not notably alter the potential of this site, 5 clashes at the atomic level were identified between Pro678 and 2 spatially adjacent amino acids, Val677 and Ile596 (Fig. S2C) [15]. This may impair the normal tertiary structure of NC1, blocking the trimerization of type X collagen.

## Literature Review

### Phenotypes of Patients with MCDS

In addition to the 4 patients above, 124 cases of MCDS have been reported in the literature, among which the clinical and radiographic manifestations were delineated in 92 cases (Table S1 [15]).

Male and female patients accounted for 54% and 46%, respectively. The median follow-up was 3.7 years (0–31 years, *n* = 34). Short stature was the most common sign (35.8%, 19/53), and the patients' height Z-scores were − 2.77 ± 1.78 at the initial examination and were − 2.98 ± 1.87 at the last evaluatio*n*. However, not all patients met the criteria of short stature. 40.0% (10/25) and 32.4% (22/68) of patients at first and last examinations, respectively, presented with a height Z-score > − 2.0. Shortened limb (95.6%, 43/45), waddling gait (88.5%, 54/61), genu varum (83.6%, 56/67), and lumbar lordosis (51.6%, 16/31) were common clinical manifestations, while genu valgum (10.4%, 7/67) was relatively rare.

On radiography, patients showed short femoral neck (81.7%, 49/60), bowed femurs or tibiae (81.5%, 44/54), coxa vara (80.0%, 60/75), and enlarged capital femoral epiphysis (77.4%, 41/53). Metaphyseal irregularities most commonly involved proximal femurs (93.3%, 56/60), followed by proximal tibiae and fibulae (93.1%, 54/58), distal femurs (91.8%, 56/61), distal tibiae and fibulae (84.9%, 45/53), and distal radius or ulna (57.6%, 19/33).

The first diagnosis of MCDS was made between 0.9 and50 years (median 3.5 years). Although MCDS was the initial diagnosis in 94.4% (117/124) of cases, 7 cases were misdiagnosed with rickets due to the shared metaphyseal enlargement, fraying, and splaying, including 3 cases with X-linked hypophosphatemic rickets, 3 cases with rickets, and 1 case with vitamin D-deficient rickets.

Treatment was mentioned in 24 cases, among which 20 (74.1%) underwent osteotomies, 4 (14.8%) received medication, including calcium, vitamin D, or rhGH. No significant difference was found in height Z-scores before and after treatment (median − 2.10, − 3.21, respectively; *P* = 0.503). A 2-year-old boy with MCDS was administered long-acting rhGH at a dose of 0.17 IU/kg once a week, combined with a daily supplement of calcium and vitamin D for 6 months [16]. His height increased from 81 to 86 cm (Z-score − 2.1 for both) after therapy.

### Genotype

*COL10A1* mutations were reported in 114 cases, of which 53.5% of the cases harbored missense variants, and 44.7% carried truncating mutations, including nonsense (16.7%) and frameshift in *COL10A1* (28.1%) (Fig. S3) [15]. 90.4% of the cases had mutations in the NC1 domain of collagen X, and the rest contained 7 cases with mutations in the NC2 domain, 5 in the signal peptide domain, 2 in the 3' UTR, and 1 in the helix domain. The origin of *COL10A1* mutations was determined in 68 cases, with 82.4% being inherited and 17.6% de novo. Zygosity of the mutations was described in 91 cases, of which 92.3% were heterozygous, while 7.7% were homozygous. The homozygous cases were considered recessively inherited. The vast majority of patients had a single *COL10A1* mutation, except two brothers who harbored the same missense mutation in the NC1 domain and a deletion in the 3′ UTR. c.1832G > A (p.Trp611*) was reported in 4 unrelated families [17, 18], which could be a hotspot mutation.

### Genotype–Phenotype Relationships

We categorized *COL10A1* mutations into 3 groups: mutations in the NC1 or non-NC1 domain, missense or truncating mutations, and missense or truncating mutations in the NC1 domain. Then, we compared the differences in clinical and radiographic manifestations of patients in the literature and PUMCH between each pair (Table 3). We found that patients with variants in the NC1 domain presented typical signs at a median age of 12 months, significantly earlier than those with non-NC1 domain variants (median 72 months, *P* = 0.0014). No significant differences in onset age were identified between patients with missense or truncating mutations, or between those with missense or truncating mutations in the NC1 domain.

**Table 3:** Genotype–phenotype relationships in MCDS cases in the literature and in PUMCH

	NC1 mutations	Non-NC1 mutations	*P* value	Missense	Truncating mutations	*P* value	NC1-missense	NC1-truncating mutations	*P* value
Age of initial sign (month)	12.0 (10.0, 24.0) (*n* = 58)	72.0 (12.0, 72.0) (*n* = 11)	0.0014	12.0 (10.0, 42.0) (*n* = 41)	12.0 (12.0, 24.0) (*n* = 25)	0.820	12.0 (8.5, 23.0) (*n* = 32)	12.0 (12.0, 24.0) (*n* = 25)	0.311
Height Z-score at first evaluation	− 2.37 ± 1.67 (*n* = 26)	− 4.18 ± 3.11 (*n* = 3)	0.115	− 3.62 ± 1.95 (*n* = 12)	− 1.81 ± 1.44 (*n* = 17)	0.008	− 3.43 ± 1.63 (*n* = 9)	− 1.81 ± 1.44 (*n* = 17)	0.015
Height Z-score at last evaluation	− 2.61 ± 1.46 (*n* = 61)	− 5.58 ± 1.95 (*n* = 10)	0.0001	− 2.20 (− 4.30, − 1.20) (*n* = 41)	− 3.00 (− 3.90, − 2.35) (*n* = 29)	0.163	− 2.04 ± 1.46 (*n* = 31)	− 3.19 ± 1.26 (*n* = 29)	0.002
Metaphyseal irregularities in the distal radius/ulna	*n*( +) = 15 *n*(−) = 17	*n*( +) = 4 *n*(−) = 0	0.106	*n*( +) = 11 *n*(−) = 3	*n*(+) = 8 *n*(−) = 14	0.019	*n*(+) = 7 *n*(−) = 3	*n*( +) = 8 *n*(−) = 14	0.128

NC, non-collagenous; *n*(+), number of patients with metaphyseal irregularities in distal radius/ulna; *n*(−), number of patients without metaphyseal irregularities in distal radius/ulna

At the first evaluation, height Z-scores of patients with missense mutations were significantly lower than those with truncating mutations of *COL10A1* (− 3.62 ± 1.95 vs. − 1.99 ± 1.28, *P* = 0.013), and the differences remained significant when restricting both mutations to the NC1 domain (− 3.43 ± 1.63 vs. − 1.99 ± 1.28, *P* = 0.022). However, no remarkable difference was found in height Z-scores at the first evaluation between patients with NC1 mutations and those with non-NC1 mutations. At the last evaluation, patients with non-NC1 mutations had significantly lower height Z-scores compared to those with NC1 mutations (− 5.58 ± 1.95 vs. − 2.61 ± 1.46, *P* = 0.0001). No significant difference was identified in the height Z-scores at last evaluation between patients with missense or truncating variants, but patients with NC1 truncating variants showed notably lower height Z-scores than those with NC1 missense (− 3.19 ± 1.26 vs. − 2.04 ± 1.46, *P* = 0.002).

We also found that the proportion of metaphyseal irregularities at the distal radius/ulna was significantly higher in patients carrying missense than those with truncating mutations (*P* = 0.019) (Table 3). However, the proportion of other manifestations did not differ significantly among the three genotype groups of patients (data not shown).

In addition, molecular results of parents were available in 76 reported cases. Among them, only the parents of 4 unrelated cases carried a *COL10A1* missense mutation in the NC1 domain and did not exhibit clinical or radiographical features of MCDS (Table S1 [15]). Patient 18 with a biallelic c.1954C > T variant shows severe short stature (height Z-score − 3.9) and lower limb deformities, while both parents heterozygous for the same variant show no abnormality on physical examination or radiography. Patient 43’s mother, who harbored the same heterozygous c.1765T > A mutation as the patient, is slightly short in height (Z-score − 1.3) without any other manifestation of MCDS. Similar findings are also observed in patient 54’s mother with a heterozygous c.1846A > G mutation. Patient 82’s mother, confirmed mosaic for the c.1771T > C variant, is unaffected with a height > 25th percentile.

## Discussion

In this study, we investigated the clinical presentation, radiographic features, and genotypes of a small cohort of rare disease MCDS caused by *COL10A1* gene mutations. We also analyzed genotype–phenotype relationships in our and previously reported MCDS cases through a careful literature review.

In our patients, the phenotypic profile mainly included waddling gait, bowed legs, short stature, and flattening or blurred vertebrae and femoral epiphysis widening in radiography. Patients usually had normal BMD and bone turnover biomarkers, accompanied by vitamin D insufficiency or deficiency. Calcium, vitamin D analogues, or GH therapy showed limited efficacy in improving the disease phenotype. *COL10A1* mutations in our patients were all heterozygous, mainly affecting the NC1 domain. Two novel *COL10A1* missense mutations, c.1925T > A (p.Ile642Asn) and c.1903C > G (p.Gln635Glu), were identified. One patient harbored a de novo NC1 domain truncating mutation and a missense mutation in the helix domain, the former possibly being mosaic, which was the first reported case of MCDS that carries mutations in both NC1 and helix domains, thereby broadening the mutational spectrum of MCDS.

MCDS is a rare disease with complex phenotypes and significant heterogeneity in clinical and radiographic manifestations. Short stature was the most common initial sign of MCDS, which persisted throughout the course of the disease. However, 40% of patients did not suffer short stature at first, suggesting the importance of other manifestations in recognizing MCDS, including shortened limbs, waddling gait, as well as short femoral neck, bowed femurs or tibiae, and metaphyseal irregularities at femurs, tibiae, and fibulae displayed on radiography. We found that 56.3% of cases also had metaphyseal irregularities at the distal radius or ulna, similar to the previous finding that wrist involvement occurred in 61.5% of cases [2]. A significant proportion of patients were initially misdiagnosed with different types of rickets, possibly due to the overlapping manifestations between the two diseases, such as bowed legs, genu varum or genu valgum, metaphyseal enlargement, fraying, and splaying [8–10]. However, MCDS usually lacks blurred trabecular bone and maintains normal serum levels of Ca, P, 25OHD, β-CTX, and P1NP, which help differentiate the two disorders [8, 19].

Type X collagen, a short-chain non-fibrillar collagen, is specifically secreted by hypertrophic chondrocytes in the growth plate into their surrounding extracellular matrix (ECM) [20, 21]. As a major component of the growth plate hypertrophic zone, it plays an important role in calcification and vascular invasion during endochondral ossification by assembling into hexamers [22]. Four domains comprise type X collagen, of which the carboxy-terminal NC1 domain is essential for the assembly of the α1(X) homotrimer, where MCDS mutations occur most frequently [16, 22, 23].

Of note, we identified an intricate relationship between the mutation type of *COL10A1* and the degree of short stature in MCDS patients. At the first examination, no significant difference in height Z-scores was found between patients with NC1 mutations and non-NC1 mutations. However, at the last evaluation, patients with non-NC1 mutations showed significantly lower height Z-scores. This is presumably because most patients with non-NC1 mutations came from the same family carrying homozygous mutations in the NC2 domain, who showed a late onset of short stature until the age of 6, with severely low height Z-scores in adulthood (− 4.8 to − 7.5) [24]. Mechanistically, in vitro functional studies have shown that mutations in the signal peptide also considerably impair collagen X assembly, leading to a reduction of normal collagen X secretion [25]. However, the influence of NC2 mutations on collagen X expression has not been explored. This finding remains to be validated with larger samples and in vitro studies in patients with MCDS.

Intriguingly, NC1-missense patients had a significantly lower height Z-score initially than patients with NC1-truncating mutations, but the opposite was true at the last assessment, indicating that short stature may progress with age in the presence of NC1-truncating variants. It has been shown that mutant *COL10A1* mRNA containing nonsense or frameshift mutations would be degraded via nonsense-mediated decay [26, 27]. In contrast, this process was not observed in *COL10A1* mRNA with a missense variant in growth plate cartilage from a patient with MCDS [28], and a trace amount of mutant/normal α1(Ⅹ) chain heterotrimer assembly was detected in vitro [29]. Thus, haploinsufficiency due to NC1-truncating mutations may partially explain this phenomenon [18, 30].

The above mechanism may also explain the reduction in penetrance. The penetrance of the *COL10A1* mutation has been reported as nearly 100% [1]. However, asymptomatic carrier parents exist in both the two families in our study and 4 unrelated families described in the literature, who showed normal or slightly short stature (Z-score > − 2.0) without other clinical or radiographical manifestations of MCDS [17, 31, 32]. Notably, most of them harbored missense but not truncating mutations in the NC1 domain. This further indicates that NC1-missense mutations exert a less pronounced impact on the skeletal phenotypes over time compared to NC1-truncating mutations. Consistently, previous studies have observed that certain radiographical manifestations resolve with age, including enlarged femoral head, platyspondyly, and metaphyseal cupping of phalanges and metacarpals [17, 33, 34]. Most of the cases also harbored missense mutations in *COL10A1*.

Furthermore, we found that the proportion of metaphyseal involvement at the distal radius or ulna was significantly higher in patients with missense or NC1 missense. No studies have yet identified an association between a specific *COL10A1* genotype and a particular epiphyseal involvement. Additional cases are required to verify this finding.

To date, no medications have been approved for the treatment of MCDS. Most of the patients with skeletal deformities, such as significant lower limb curvature, received orthopedic surgery, but the height Z-score did not improve greatly after treatment. Previous studies showed that rhGH therapy modestly enhanced the height Z-score of MCDS patients during 0.5–2.25 years [16, 35]. However, our study showed that GH treatment did not significantly increase the height of patients and could not induce catch-up growth, possibly because GH therapy is unable to reverse the effects of *COL10A1* mutations on bone growth [16, 35].

To find therapeutic targets for MCDS, the pathogenesis has been partially revealed. Mutant collagen is retained intracellularly, triggering endoplasmic reticulum (ER) stress and unfolded protein response, which in turn may compromise chondrocyte differentiation and polarity, leading to disorganized cell stacking and ultimately growth plate irregularities [21, 36, 37]. Interestingly, carbamazepine (CBZ), an anti-epileptic drug, has been recently repurposed to treat in vitro and in vivo models of MCDS due to its inhibitory effects on ER stress by enhancing autophagy and proteosomal degradation [21, 36, 38, 39]. In 3-week-old MCDS mice with *Col10a1* p.N617K mutation, 3-week CBZ treatment (250 mg/kg/d) significantly reduced the width of the hypertrophic zone, increased femur and tibia growth, and alleviated hip dysplasia [21]. The same effects of CBZ were also observed in mice harboring *Col10a1* p.Y632X mutation and medaka model with a *Col10a1* frameshift at amino acid 633 [36, 39]. Mechanistically, CBZ treatment inhibited ER stress-related gene expression, including Bip, Atf4, and attenuated disruption of type X collagen expression [21, 39]. Meanwhile, CBZ restored the polarity of hypertrophic chondrocytes and the coordinated expression of their differentiation markers, Opn and Mmp13 [21, 36]. These may ameliorate the damaged growth plate, thereby restoring normal bone growth. More recently, phase 1 and phase 2/3 clinical trials of CBZ treatment on 27 children and adolescents with MCDS are ongoing in Europe [38, 40]. The participants received CBZ at a maximum dose of 20 mg/kg/day for at least 12 months [40]. Periodic reporting claims that CBZ treatment is tolerated in children with MCDS and is associated with reduced bone pain, accelerated growth, and decreased progression of deformity in the lower extremities [41]. However, long-term use of CBZ increases the risk of osteoporosis and fractures through inducing CYP24, a catabolic enzyme for 1,25(OH)_2_D, which accelerates 1,25(OH)_2_D inactivation [42, 43]. Therefore, the safety of CBZ for long-term treatment in MCDS patients remains to be evaluated.

However, our study has some limitations. First, some of the reported cases did not provide clinical manifestations in detail or undergo molecular testing, inevitably impairing the solidity of the results. Secondly, cartilage specimens were not obtained from patients to analyze the ratio of normal to mutant collagen X. Functional validation of the novel variants was not performed to rationalize the genotype–phenotype correlation. Additionally, we were unable to obtain the radiography of the unaffected carriers in families 1 and 2 to adequately explain the asymptomatic condition.

In conclusion, our findings expand the mutational and phenotypic spectrum of MCDS and shed new light on the relationships between *COL10A1* mutation and the severity of MCDS, emphasizing the role of molecular typing in individualized assessment of MCDS patients. This study is of great significance for understanding the key role of collagen X in bone growth and development. Further studies will utilize larger-scale cases, combined with in vitro and in vivo experiments, to demonstrate the genotype–phenotype association and to develop safe and effective therapeutic approaches for patients with MCDS.

## Supplementary Information

Below is the link to the electronic supplementary material.

Supplementary Material 1

Supplementary Material 1

## Data Availability

The authors confirm that the data supporting the findings of this study are available within the article and its supplementary materials.

## References

[1] Richmond CM, Savarirayan R (2019) Schmid metaphyseal chondrodysplasia. In: Adam MP, Feldman J, Mirzaa GM et al (eds) GeneReviews®. University of Washington, Washington 31633898

[2] Lachman RS, Rimoin DL, Spranger J (1988) Metaphyseal chondrodysplasia, Schmid type. Clinical and radiographic delineation with a review of the literature. Pediatr Radiol 18(2):93–102 3281118 10.1007/BF02387549

[3] Mäkitie O, Susic M, Ward L et al (2005) Schmid type of metaphyseal chondrodysplasia and COL10A1 mutations--findings in 10 patients. Am J Med Genet A 137A(3):241–248 16088909 10.1002/ajmg.a.30855

[4] Warman ML, Abbott M, Apte SS et al (1993) A type X collagen mutation causes Schmid metaphyseal chondrodysplasia. Nat Genet 5(1):79–82 8220429 10.1038/ng0993-79

[5] Bonaventure J, Chaminade F, Maroteaux P (1995) Mutations in three subdomains of the carboxy-terminal region of collagen type X account for most of the Schmid metaphyseal dysplasias. Hum Genet 96(1):58–64 7607655 10.1007/BF00214187

[6] Gu J, Lu Y, Li F et al (2014) Identification and characterization of the novel Col10a1 regulatory mechanism during chondrocyte hypertrophic differentiation. Cell Death Dis 5(10):e1469 25321476 10.1038/cddis.2014.444 PMC4649528

[7] Wallis GA, Rash B, Sykes B et al (1996) Mutations within the gene encoding the alpha 1 (X) chain of type X collagen (COL10A1) cause metaphyseal chondrodysplasia type Schmid but not several other forms of metaphyseal chondrodysplasia. J Med Genet 33(6):450–457 8782043 10.1136/jmg.33.6.450 PMC1050629

[8] Chen Q, Wu S-N, Chen Y-X et al (2020) A novel missense COL10A1 mutation: c.2020G>A; p. Gly674Arg linked with the bowed legs stature in the Schmid metaphyseal chondrodysplasia-affected Chinese lineage. Bone Rep 12:100240 31921940 10.1016/j.bonr.2019.100240 PMC6950639

[9] Goyal M, Gupta A, Choudhary A et al (2019) Schmid type metaphyseal chondrodysplasia with a novel COL10A1 mutation. Indian J Pediatr 86(2):183–185 30209734 10.1007/s12098-018-2791-0

[10] Wallis GA, Rash B, Sweetman WA et al (1994) Amino acid substitutions of conserved residues in the carboxyl-terminal domain of the alpha 1(X) chain of type X collagen occur in two unrelated families with metaphyseal chondrodysplasia type Schmid. Am J Hum Genet 54(2):169–178 8304336 PMC1918153

[11] Hui L, Cheng-ye J, Xin-nan Z et al (2009) Height and weight standardized growth charts for Chinese children and adolescents aged 0 to 18 years. Chin J Pediatr 47(7):487–492 19951507

[12] Khadilkar AV, Sanwalka NJ, Chiplonkar SA et al (2011) Normative data and percentile curves for Dual Energy X-ray Absorptiometry in healthy Indian girls and boys aged 5–17 years. Bone 48(4):810–819 21182992 10.1016/j.bone.2010.12.013

[13] Xu H, Zhao Z, Wang H et al (2013) Bone mineral density of the spine in 11,898 Chinese infants and young children: a cross-sectional study. PLoS ONE 8(12):e82098 24324752 10.1371/journal.pone.0082098 PMC3855755

[14] Richards S, Aziz N, Bale S et al (2015) Standards and guidelines for the interpretation of sequence variants: a joint consensus recommendation of the American College of Medical Genetics and Genomics and the Association for Molecular Pathology. Genet Med 17(5):405–424 25741868 10.1038/gim.2015.30 PMC4544753

[15] Meng L, Hu J, Sun L, et al (2025) Supplementary data for: clinical, molecular characteristics and genotype-phenotype relationships of metaphyseal chondrodysplasia type Schmid. figshare. 10.6084/m9.figshare.30039370.v2. Deposited 17 September 2025 10.1007/s00223-025-01457-8 PMC12743679 41454937

[16] Wu H, Wang S, Li G et al (2021) Characterization of a novel COL10A1 variant associated with Schmid-type metaphyseal chondrodysplasia and a literature review. Mol Genet Genomic Med 9(5):e1668 33764685 10.1002/mgg3.1668 PMC8172203

[17] Tüysüz B, Kasap B, Sarıtaş M et al (2023) Natural history and genetic spectrum of the Turkish metaphyseal dysplasia cohort, including rare types caused by biallelic COL10A1, COL2A1, and LBR variants. Bone 167:116614 36400164 10.1016/j.bone.2022.116614

[18] Bateman JF, Freddi S, Nattrass G et al (2003) Tissue-specific RNA surveillance? Nonsense-mediated mRNA decay causes collagen X haploinsufficiency in Schmid metaphyseal chondrodysplasia cartilage. Hum Mol Genet 12(3):217–225 12554676 10.1093/hmg/ddg054

[19] Vohra P (1996) Metaphyseal chondrodysplasia—a differential diagnosis of rickets. Indian J Pediatr 63(1):127–128 10829978 10.1007/BF02823884

[20] Ağırdil Y (2020) The growth plate: a physiologic overview. EFORT Open Rev 5(8):498–507 32953135 10.1302/2058-5241.5.190088 PMC7484711

[21] Mullan LA, Mularczyk EJ, Kung LH et al (2017) Increased intracellular proteolysis reduces disease severity in an ER stress-associated dwarfism. J Clin Invest 127(10):3861–3865 28920921 10.1172/JCI93094 PMC5617653

[22] Bateman JF, Wilson R, Freddi S et al (2005) Mutations of COL10A1 in Schmid metaphyseal chondrodysplasia. Hum Mutat 25(6):525–534 15880705 10.1002/humu.20183

[23] Bateman JF, Freddi S, McNeil R et al (2004) Identification of four novel COL10A1 missense mutations in schmid metaphyseal chondrodysplasia: further evidence that collagen X NC1 mutations impair trimer assembly. Hum Mutat 23(4):396 15024737 10.1002/humu.9222

[24] Ain Nu, Makitie O, Naz S (2018) Autosomal recessive chondrodysplasia with severe short stature caused by a biallelic COL10A1 variant. J Med Genet 55(6):403–407 28830906 10.1136/jmedgenet-2017-104885

[25] Chan D, Ho MS, Cheah KS (2001) Aberrant signal peptide cleavage of collagen X in Schmid metaphyseal chondrodysplasia. Implications for the molecular basis of the disease. J Biol Chem 276(11):7992–7997 11115494 10.1074/jbc.M003361200

[26] Tan JT, Kremer F, Freddi S et al (2008) Competency for nonsense-mediated reduction in collagen X mRNA is specified by the 3’ UTR and corresponds to the position of mutations in Schmid metaphyseal chondrodysplasia. Am J Hum Genet 82(3):786–793 18304492 10.1016/j.ajhg.2008.01.006 PMC2427218

[27] Yang J, Zhang J, Lu Q et al (2025) The p.W651fsX666 mutation on COL10A1 results in impaired trimerization of normal collagen X to induce Schmid type Metaphyseal chondrodysplasia. Hum Mol Genet 34(15):1265–1285 40398448 10.1093/hmg/ddaf071

[28] Gregory CA, Zabel B, Grant ME et al (2000) Equal expression of typ X collagen mRNA fom mutant and wild type COL10A1 alleles in growth plate cartilage from a patient with metaphyseal chondrodysplasia type Schmid. J Med Genet 37(8):627–629 10991694 10.1136/jmg.37.8.627 PMC1734661

[29] Wilson R, Freddi S, Bateman JF (2002) Collagen X chains harboring Schmid metaphyseal chondrodysplasia NC1 domain mutations are selectively retained and degraded in stably transfected cells. J Biol Chem 277(15):12516–12524 11805116 10.1074/jbc.M112044200

[30] Chan D, Weng YM, Graham HK et al (1998) A nonsense mutation in the carboxyl-terminal domain of type X collagen causes haploinsufficiency in schmid metaphyseal chondrodysplasia. J Clin Invest 101(7):1490–1499 9525992 10.1172/JCI1976 PMC508727

[31] Kong L, Shi L, Wang W et al (2019) Identification of two novel COL10A1 heterozygous mutations in two Chinese pedigrees with Schmid-type metaphyseal chondrodysplasia. BMC Med Genet 20(1):200 31856751 10.1186/s12881-019-0937-1 PMC6923838

[32] McIntosh I, Abbott MH, Warman ML et al (1994) Additional mutations of type X collagen confirm COL10A1 as the Schmid metaphyseal chondrodysplasia locus. Hum Mol Genet 3(2):303–307 8004099 10.1093/hmg/3.2.303

[33] Elliott AM, Field FM, Rimoin DL et al (2005) Hand involvement in Schmid metaphyseal chondrodysplasia. Am J Med Genet A 132A(2):191–193 15578582 10.1002/ajmg.a.30433

[34] Savarirayan R, Cormier-Daire V, Lachman RS et al (2000) Schmid type metaphyseal chondrodysplasia: a spondylometaphyseal dysplasia identical to the “Japanese” type. Pediatr Radiol 30(7):460–463 10929364 10.1007/s002470000181

[35] Chen M, Miao H, Liang H et al (2022) Clinical characteristics of short-stature patients with collagen gene mutation and the therapeutic response to rhGH. Front Endocrinol (Lausanne) 13:820001 35250876 10.3389/fendo.2022.820001 PMC8889571

[36] Tan WH, Rücklin M, Larionova D et al (2024) A Collagen10a1 mutation disrupts cell polarity in a medaka model for metaphyseal chondrodysplasia type Schmid. iScience 27(4):109405 38510140 10.1016/j.isci.2024.109405 PMC10952040

[37] Rajpar MH, McDermott B, Kung L et al (2009) Targeted induction of endoplasmic reticulum stress induces cartilage pathology. PLoS Genet 5(10):e1000691 19834559 10.1371/journal.pgen.1000691 PMC2757901

[38] Sabir AH, Cole T (2019) The evolving therapeutic landscape of genetic skeletal disorders. Orphanet J Rare Dis 14(1):300 31888683 10.1186/s13023-019-1222-2 PMC6937740

[39] Forouhan M, Sonntag S, Boot-Handford RP (2018) Carbamazepine reduces disease severity in a mouse model of metaphyseal chondrodysplasia type Schmid caused by a premature stop codon (Y632X) in the Col10a1 gene. Hum Mol Genet 27(22):3840–3853 30010889 10.1093/hmg/ddy253 PMC6216233

[40] Beacon for rare diseases. MCDS-Therapy|EU Horizon 2020 project |Rare bone disease. Accessed August 10, 2025. https://www.rarebeacon.org/research/mcds-therapy/

[41] CORDIS - EU research results. Repurposing of carbamazepine for treatment of skeletal dysplasia. Updated February 19, 2025. Accessed August 10, 2025. https://cordis.europa.eu/project/id/754825/reporting

[42] Brodie MJ, Mintzer S, Pack AM et al (2013) Enzyme induction with antiepileptic drugs: Cause for concern? Epilepsia 54(1):11–27 23016553 10.1111/j.1528-1167.2012.03671.x

[43] Dussault PM, McCarthy D, Davis SA et al (2021) High prevalence of vertebral fractures in seizure patients with normal bone density receiving chronic anti-epileptic drugs. Osteoporos Int 32(10):2051–2059 33822290 10.1007/s00198-021-05926-2

