# Supplementary material for: Clinical, Molecular Characteristics, and Genotype–Phenotype Relationships of Metaphyseal Chondrodysplasia Type Schmid
Source: Calcif Tissue Int. 2025 Dec 27;117(1):5. doi: 10.1007/s00223-025-01457-8 (PMC12743679; doi:10.1007/s00223-025-01457-8)
Supplement: Supplementary file 1 — Supplementary Material 1 [file 223_2025_1457_MOESM1_ESM.docx]

**Supplemental Material and Methods**

**Sanger sequencing**

The forward and reverse primer sequences of *COL10A1* variants identified in 4 patients with MCDS in PUMCH are listed below:

| **Primer name** | **Primer sequence** |
| --- | --- |
| COL10A1-c.2032-F | GGGAAGGTTTGTTGGTCTGATAG |
| COL10A1-c.2032-R | TCAGGGAGTGCCATCATCG |
| COL10A1-c.1438-F | AGGGAATGCCCGGACACAAT |
| COL10A1-c.1438-R | GTTGGCACTAACAAGAGGGGT |
| COL10A1-c.2001-F | CGTGCATGTGAAAGGGACTC |
| COL10A1-c.2001-R | TCAGGGGGAAGGTTTGTTGG |
| COL10A1-c.1925-F | TTTTCAGCCTACCTCCATATGC |
| COL10A1-c.1925-R | AACAGCATTATGACCCAAGGAC |

**Supplementary Figure S1**

**
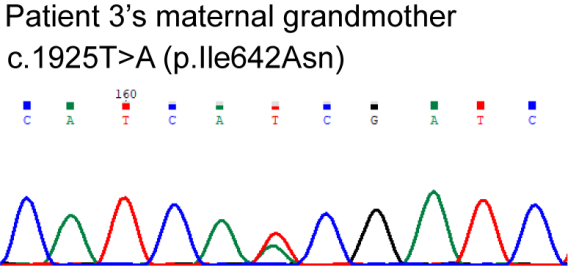
**

**Figure S1.** Sanger sequencing of *COL10A1* mutation in patient 3’ maternal grandmother.

**Supplementary Figure S2**


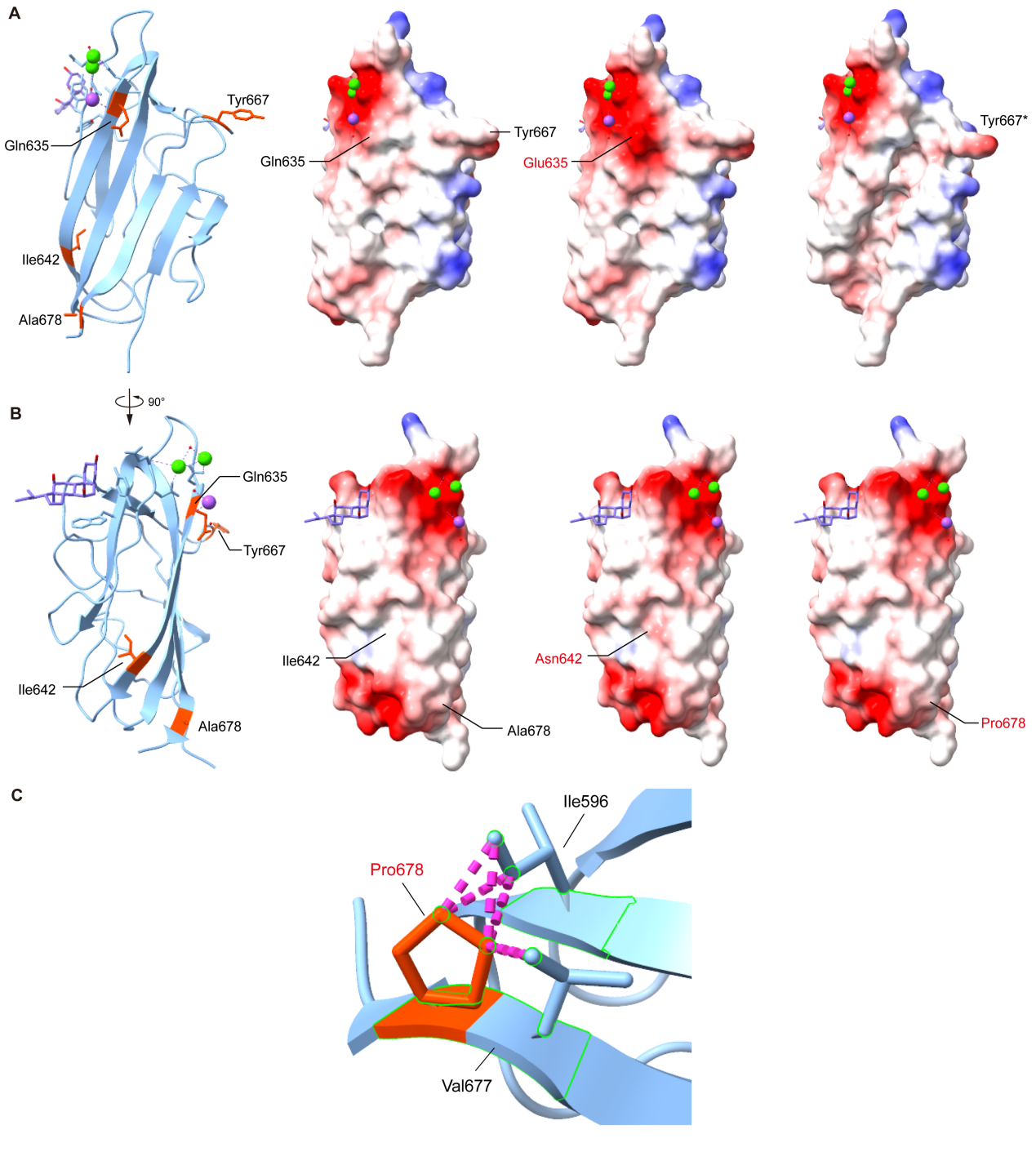


**Figure S2.** Position and potential changes of NC1 domain mutations.

1. Position and corresponding electrostatic potential changes of NC1 domain mutations in patients 2 and 4. Tyr667* in patient 2 leads to the absence of a peptide segment. Gln635Glu in patient 4 enhances the negative potential.

(B) Position and corresponding electrostatic potential changes of NC1 domain mutations in patients 1 and 3. Ala678Pro in patient 1 did not notably change the potential. Ile642Asn in patient 3 enhances the negative potential. (B) is obtained from (A) rotating by 90°.

(C) Amino acid spatial conflict analysis of the Ala678Pro mutation in patient 1. Purple dashed lines represent atomic clashes between adjacent amino acids. Black and red text indicate wild-type and mutant amino acids, respectively.

**Supplementary Figure S3**


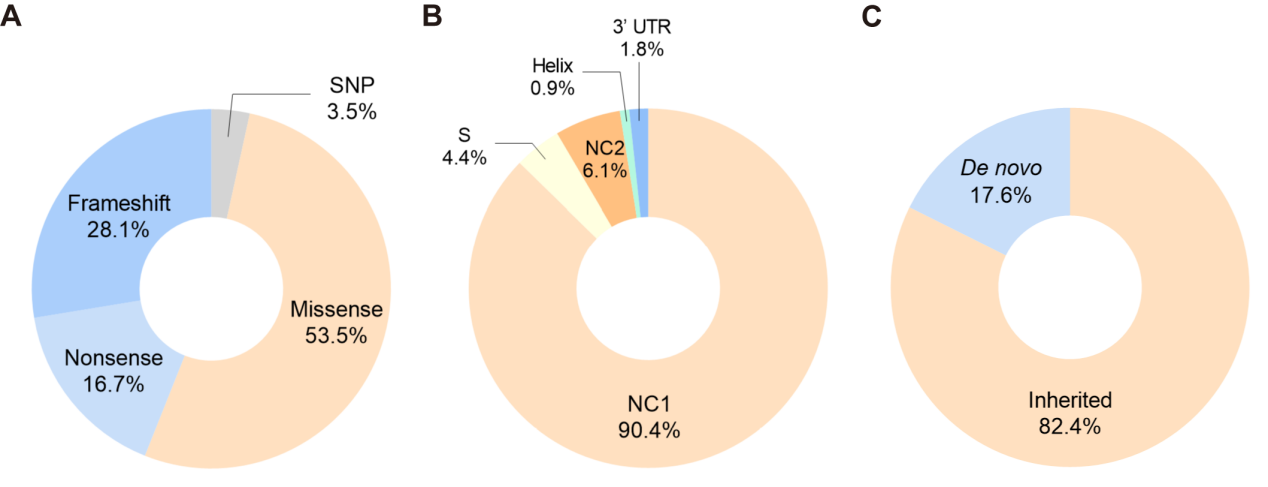


**Figure S3.** Genotype categories in MCDS cases in the literature.

1. Percentage of missense, nonsense, framshift, and SNP of the *COL10A1* gene in MCDS cases. SNP, single nucleotide polymorphism.

(B) Percentage of mutations in different domains of collagen X. NC, non-collagenous; S, signal peptide; UTR, untranslated region.

(C) Percentage of inherited and *de novo* *COL10A1* mutations in MCDS cases.
